# Working With a Psychopath: Is There Light at the End of the Tunnel?

**DOI:** 10.1177/0306624X211058957

**Published:** 2021-11-20

**Authors:** Jayme Stewart, Adelle Forth, Janelle Beaudette

**Affiliations:** 1 Carleton University, Ottawa, ON, Canada; 2 Public Safety Canada, Ottawa, ON, Canada

**Keywords:** corporate psychopathy, psychopathy, victimization, posttraumatic growth, resiliency

## Abstract

Having a supervisor with psychopathic characteristics is related to being bullied, poorer job satisfaction, work/family life conflict, financial instability, and distress. To date, all research on corporate psychopathy victims considers how they are negatively impacted rather than potential positive outcomes. In response, this study examined how working with a psychopath impacts posttraumatic growth (PTG). Utilizing a mixed-methods approach, this study draws upon the experiences of 285 individuals who have worked with a colleague or supervisor with alleged psychopathic characteristics. Results indicated that approach coping and psychopathic characteristics predicted PTG. Qualitative analyses revealed that the majority of participants used various coping strategies (e.g., emotion-focused), received support (e.g., emotional), and underwent post-experiential growth or learning (e.g., positive personal growth); not all growth/learning was positive, however (e.g., less trusting). Results suggest that cultivating approach-focused coping strategies may enhance PTG following a traumatic event.

## Introduction

Psychopathy is a personality disorder comprised of a constellation of affective (e.g., callousness, lack of remorse), interpersonal (e.g., grandiose sense of self-worth, manipulation), lifestyle (impulsivity, parasitic lifestyle), and antisocial (poor anger control, early behavioral problems; Hare, 1996; Hare & Neumann, 2008) characteristics that is often associated with malevolent and socially malicious behaviors (Paulhus & Williams, 2002). Over the last few decades, the role of psychopathy within maladaptive behaviors such as delinquency and crime has been studied extensively, with research demonstrating that psychopathic individuals are criminally versatile (e.g., engaging in frequent and diverse violent and non-violent crimes; Blackburn & Coid, 1998; Fix & Fix, 2015; Olver & Wong, 2006; Porter et al., 2001, 2009). More recently, the notion of the “successful psychopath,” or one that has avoided maladaptive consequences (e.g., arrests) and has obtained desirable outcomes in life (e.g., successful employment; Benning et al., 2018), has become an avenue of research for many, where researchers have predominately explored the effects of psychopathy in the workplace. Most notably, research has considered how survivors of corporate psychopaths are negatively impacted rather than any potential positive outcomes. In response, this study examined how working with an alleged psychopath impacts posttraumatic growth (PTG).

In a recent survey of over 5,000 companies worldwide, 47% indicated experiencing fraud within the past 24 months, with losses totaling $42 billion USD (PricewaterhouseCoopers, 2020). Forty-three percent of those recounting losses of $100 million USD were reported to be committed by middle or senior management, and operations staff (PricewaterhouseCoopers, 2020). Tepper et al. (2006) estimated that in the United States, corporations lose approximately $23.8 billion annually due to issues related to productivity or health care costs resultant from abusive supervisory styles. Abusive leadership is related to decreases in creativity (Liu et al., 2012), performance (Harris et al., 2007), job satisfaction, and organizational commitment (Tepper, 2000), while simultaneously related to feelings of distress (Tepper, 2000), increases in work/family conflict (Tepper, 2000), and workplace deviance or job neglect (i.e., spending less time doing work and more time on non-work activities [e.g., breaks]; Lim, 2002; Mitchell & Ambrose, 2007; Tepper et al., 2008).

Across numerous mono-method studies using other-ratings of both psychopathic characteristics and criterion-related outcomes (e.g., work-related behaviors/experiences), in addition to employing varied measures of psychopathic characteristics (e.g., B-SCAN, Mathieu et al., 2013; Dirty Dozen, Jonason & Webster, 2010; Psychopathy Measure—Management Research Version, Boddy, 2010), psychopathy in the workplace has, unsurprisingly, demonstrated to be positively associated with abusive supervisory styles (Boddy, 2011; Mathieu & Babiak, 2016) and appears to be a better predictor of employee satisfaction and attitudes than supervisory style alone (Mathieu & Babiak, 2015). Babiak et al. (2010) found that despite receiving poor management or performance appraisals (e.g., being a team player, accomplishments) by their superior, psychopathic characteristics, as assessed by a rater trained in the Psychopathy Checklist—Revised (Hare, 2003), were related to being perceived as having leadership potential. Spencer and Byrne (2016) reported that those with psychopathic characteristics are more heavily represented in senior-ranking positions than lower-level or entry positions. Certainly, given the myriad of negative characteristics often exhibited and associated with psychopathy, the potential implications of employing an individual with psychopathic characteristics, particularly within managerial positions, may have serious consequences to employees’ mental and physical well-being. In addition, employing an individual with psychopathic characteristics may contribute to potential losses in revenue attributable to behaviors including employee turnover, decreased productivity or fraud.

Indeed, those working under or alongside an individual with psychopathic characteristics have reported experiencing bullying (Boddy, 2011) psychological distress and negative well-being (Boddy, 2014; Mathieu et al., 2014; Volmer et al., 2016), lesser career success (e.g., salary, promotions; Volmer et al., 2016), more work-family conflict (Mathieu et al., 2014), higher turn-over intentions (e.g., plans to leave the organization; Mathieu & Babiak, 2015, 2016), work neglect (Mathieu & Babiak, 2015), and lower overall job satisfaction (Mathieu & Babiak, 2015, 2016; Mathieu et al., 2014; Volmer et al., 2016). In his study examining the relationship between corporate psychopathy, conflict, and employee well-being, Boddy (2014) found that in comparison to when corporate psychopathy was not present, when present within an organization, instances of bullying and conflict (e.g., getting into arguments with others) occurred more frequently and aggressively, and employees were more likely to report feelings of unease, anger, anxiousness, depression, and discouragement.

While previous studies have reported negative effects in the workplace when individuals with high levels of psychopathic characteristics are present, not all experiences of traumatic or stressful events result in deleterious psychological outcomes. In the face of adversity, some individuals may undergo positive effects such as feelings of growth or resiliency. Indeed, how one perceives and processes a traumatic or stressful event may play an important role in whether one reports positive or negative changes following that event. Negatively perceiving a traumatic event may maintain symptoms related to PTSD (e.g., distress, anxiety, depression; Ehlers & Clark, 2000), while being able to positively appraise adverse events may result in positive well-being, lower depression and other beneficial outcomes such as personal strength and improved relationships (Helgeson et al., 2006; Tedeschi & Calhoun, 1996; Widows et al., 2005). Further, there is research to suggest that experiencing distress shortly before or after a traumatic event (e.g., violence, loss of a loved one, health problems) may result in positive personal growth even years after the event has occurred (Kunst, 2010; Yilmaz & Zara, 2016). This experience of exhibiting positive outcomes as a result of adverse events is often referred to as “posttraumatic growth” (PTG).

Shigemoto et al. (2017) found that being able to foster positive personal changes by identifying areas in which growth can occur, was positively correlated with PTG. Furthermore, in their study utilizing a sample of intimate partner violence (IPV) survivors, Valdez and Lilly (2005) reported that following an act of IPV, a woman’s view that the world is predictable, controllable, and generally good may be shattered, but when women were able to restructure their worldview back into a more positive schema, higher scores of PTG followed. In addition to cognitive restructuring, other methods of coping as well as social support appear to positively impact PTG. For example, He et al. (2013) noted that even after accounting for factors including social support, gender, age, and health problems, coping was a significant predictor of PTG among survivors of an earthquake. Similarly, Yilmaz and Zara (2016), found that in addition to being positively related to social support, PTG following the loss of a loved one was significantly related to engaging in approach (e.g., problem-focused) coping styles. Both perceived social support and problem-focused coping have demonstrated to be adaptive mechanisms in responding to stressful and distressing situations, where positive outcomes like improved well-being may occur (Folkman, 1997; Wethington & Kessler, 1986). Alternatively, a lack of social support or engaging in avoidance-focused coping may lead to greater feelings of distress among other unfavorable outcomes (e.g., depression, PTSD; Elklit, 2002; Endler & Parker, 1990; Kocot & Goodman, 2003).

Although there is an abundance of research geared toward studying the positive and negative outcomes that people may experience following traumatic exposure, few studies have explored the somatic and psychological consequences of those who have become involved with someone with high levels of psychopathic characteristics despite the harmful and potentially damaging effects this person may have on their victims. Of the literature available, survivors have reported experiencing economic, physical and emotional abuse, PTSD, interpersonal difficulties, trust issues, and symptoms related to depression and anxiety including insomnia, changes in eating patterns, irritability, and a lack of interest in activities previously found enjoyable (Brown & Leedom, 2008; Humeny et al., 2021; Kirkman, 2005; Pagliaro, 2009). To our knowledge however, there are no studies examining the potential positive outcomes of being associated with a psychopath in either a professional or personal capacity. The purpose of this study then was to examine, using a mixed-methods approach, the potential positive outcomes of corporate psychopathy. That is, how working with an individual with perceived psychopathic characteristics impacts employees’ post-traumatic growth and reported well-being.

At the quantitative level, research has shown that those working alongside an alleged psychopath report experiencing psychological distress, negative well-being, and increased interpersonal conflict (e.g., Mathieu et al., 2014; Volmer et al., 2016). Given this, it is possible that this group of individuals negatively perceive their experiences, potentially limiting their ability to foster positive personal growth. As such, it was expected that higher psychopathy scores would be negatively related to PTG. Alternatively, as research has demonstrated the positive relationships between coping, social support and PTG (e.g., He et al., 2013; Yilmaz & Zara, 2016), it was hypothesized that approach coping and perceived social support would be predictors of PTG such that higher scores on both approach coping and perceived social support will be related to higher PTG. At the qualitative level, it was expected that a wide range differences would emerge in how employees perceived their experiences.

## Method

### Participants

Two-hundred and eighty-five participants were selected from an initial sample of 346 participants who completed the Self-Report Psychopathy Scale—Short Form^1^ on behalf of their alleged psychopathic colleague or supervisor. Of the 285 participants, 71.6% were women (*M_age_* = 40.97, *SD_age_* = 13.35, range_age_ = 35–75) living in the United States (68.1%). Participants predominantly reported being middle-class (76.5%) and working in the medical or governmental sector (25.3%), where the majority of participants held either junior level (30.2%) or middle management positions (29.5%).

Participants were most likely to indicate working with an alleged psychopath for 1 to 2 years (31.9%), and at the time of the survey, most indicated no longer working with the alleged psychopath (63.9%). Participants described working with both men (57.2%) and women (38.6%) of primarily Caucasian descent (80.0%), where the majority of participants indicated working with an alleged psychopathic co-worker (44.2%), followed by a superior (43.5%), and subordinate (5.3%). See Table 1 for a further breakdown of participant occupational information.

**Table 1.** Participant Occupational Information.

	*n*	%
Location		
Canada	28	9.8
United States	194	68.1
Europe (UK)	19	6.7
Europe (non-UK)	18	6.3
Occupation information		
Technology/computing	32	11.2
Service/support	56	19.6
Engineering/science	21	7.4
Medical/government	72	25.3
Student	28	9.8
Position		
Entry level	70	24.6
Junior level	86	30.2
Middle management	84	29.5
Senior management	38	13.3
Length of working with alleged psychopath		
Less than a year	44	15.4
1–2 years	91	31.9
3–4 years	68	23.9
5–7 years	37	13.0
8+ years	37	13.0

### Measures

#### Posttraumatic Growth Scale

The Posttraumatic Growth Inventory (PTGI; Tedeschi & Calhoun, 1996) is a 21-item self-report measure used to assess the positive outcomes resultant of experiencing a traumatic event or crisis. In the present study, participants were advised to consider the degree to which they experienced each item as a result of working with the alleged psychopath. Participants were asked to respond to every item using a 6-point scale ranging from 0 (“*I did not experience this change as a result of working with the psychopath*”) to 5 (“*to a very great extent*”). The scale produces scores for its five subscales (i.e., new possibilities, relating to others, personal strength, spiritual change, and appreciation of life) as well as a total score. The PTGI has been validated with several populations across a variety of traumatic events (Morris et al., 2005; Tedeschi & Calhoun, 1996), and multiple languages (Jaarsma et al., 2006; Lamela et al., 2014; Pajón et al., 2020). The internal consistency for this scale utilizing all items was excellent (α = .95).

#### Perceived Support Scale

Based on the Interpersonal Support Evaluation List (ISEL; Cohen & Hoberman, 1983), the Perceived Support Scale (PSS; Kaniasty, 1988) is a 12-item questionnaire used to assess levels of perceived support obtained by victims of crime, where respondents were asked to indicate the extent to which they agree with a statement using a 4-point scale ranging from 0 (“*definitely not true*”) to 3 (“*definitely true*”). The scale provides a total perceived social support rating as well as scores for its three comprising subscales: emotional support, informational support, and tangible support. Though the PSS is intended to be used in samples of victims of crime, its parent measure, the ISEL has been used in more general contexts including depression and suicidal attempts (Harrison et al., 2010), bereavement-related complicated grief (Ghesquiere et al., 2017), and natural disasters (Norris & Kaniasty, 1996). The ISEL has demonstrated strong internal consistency and convergent validity (Ghesquiere et al., 2017; Merz et al., 2014). For this study, the internal consistency of the PSS total score was excellent (α = .91).

#### Brief COPE

The Brief COPE (Carver, 1997) is a 28-item self-report questionnaire that is used to assess coping strategies. The Brief COPE encompasses two factors (i.e., avoidant and approach coping) and 14-subscales (self-distraction, active coping, denial, substance use, emotional support, use of informational support, behavioral disengagement, venting, positive reframing, planning, humor, acceptance, religion, and self-blame), where humor and religion do not load on to either factor. Participants responded to each item using a 4-point scale ranging from 1 (“*not at all*”) to 4 (“*a lot*”). The Brief COPE has demonstrated good reliability and validity across various populations, cultures, and languages (Carver, 1997; Cooper et al., 2008; Garcia et al., 2018). The internal consistency of the Brief COPE for the present study was good (Cronbach’s α ranged from .73 [avoidance] to .84 [approach]).

#### SRP 4:SF

The Self-Report Psychopathy Scale 4th Edition: Short Form (SRP 4:SF; Paulhus et al., 2016) is a 29-item self-report questionnaire used to assess psychopathic characteristics in community, university and offender samples. Respondents were asked to complete the scale on behalf of their alleged psychopathic coworker or supervisor using a 5-point scale ranging from 1 (“*strongly disagree*”) to 5 (“*strongly agree*”). The use of a third-party perspective to assess psychopathic characteristics has been used in many studies of corporate psychopathy (e.g., Boddy, 2011, 2014; Mathieu & Babiak, 2015, 2016; Mathieu et al., 2014). Humeny et al. (2021) used the SRP 4:SF to assess psychopathic characteristics in the partner of men and women who self-identified as having a psychopathic intimate partner.

The SRP 4:SF yields four facet scores (i.e., interpersonal, affective, lifestyle, antisocial), two factor scores (Factor 1: interpersonal, affective; Factor 2: lifestyle, antisocial), and a total score (Mahmut et al., 2011). The SRP 4:SF demonstrates excellent reliability and convergent validity with other measures of psychopathy and external correlates in student, offender, and community samples (Dotterer et al., 2017; Gordts et al., 2017; Mossière et al., 2020; Seara-Cardoso et al., 2020). For this study, the internal consistency of the SRP 4:SF total score was .82.

#### Open-ended questions

Respondents were asked a series of open-ended question inquiring about their experiences of working with an alleged psychopathic colleague or superior. Specifically, the questions were as follows:

(1) How did you receive support from friends, family, or colleagues when dealing with the psychopath?
(2) How did you try to deal with the behaviors caused by the psychopath?
(3) Do you feel you’ve grown or learned important lessons as a result of having worked with the psychopath?

### Procedure

Participants were recruited from LinkedIn Canada, websites for survivors of individuals with psychopathic characteristics (Love Fraud, The Aftermath: Surviving Psychopathy Foundation, Dr. Robert Hare’s website), and Mechanical Turk. Participants on LinkedIn Canada and psychopathy related sites viewed a study advertisement seeking individuals who suspected they worked with “a psychopath,” and if interested, were directed to the online study. Alternatively, Mechanical Turk participants who met the inclusion criteria (i.e., have worked with an individual they believed to possess psychopathic characteristics) were invited to partake in the study. All interested and eligible participants completed informed consent, followed by the SRP:SF, demographic information, remaining questionnaires, and open-ended questions. Upon completion, all participants were debriefed, concluding the study.

The majority of the participants came from Mechanical Turk (31%, *n* = 88) with others coming from a variety of other web-based psychopathy related sites (e.g., LinkedIn Canada, Love Fraud, The Aftermath: Surviving Psychopathy Foundation, Dr. Hare’s website). This project was part of a larger study on the impact of psychopathy in the workforce and participants completed the measures on Survey Monkey.

### Data Analysis

#### Quantitative analysis

All data were first screened for outliers and deviations from normality and homoscedasticity, to which no issues were identified. Next, as each participant filled out the measure of psychopathy on behalf of their colleague or supervisor, it is likely that participants were not privy to all information needed to make complete or accurate judgements which may have inhibited their ability to respond to all questions on the SRP 4:SF. In response to this, participants were provided with the option of selecting “I don’t know” to any of the items they were unsure of (e.g., questions related to criminality). To account for the possibility that some participants may not be familiar with or understand the concept of psychopathy, as well as the likelihood that a participant just dislikes their co-worker/ supervisor, total scores were used to remove any participant with low psychopathy scores. This was done to ensure we were sampling from our intended population, that is, those who had worked with an individual with psychopathic characteristics. Total scores that fell between 0 and 57 were classified as low, scores that ranged between 58 and 115 were classified as moderate, and those scoring between 116 and 145 were designated as high scorers. After removal of all low scorers (*n* = 59), the final sample comprised of 285 participants.

#### Qualitative analysis

To facilitate qualitative data interpretation, content analysis was employed as this method allows for organization and categorization of participant responses in a dynamic fashion. In utilizing a dynamic process, researchers are able to search for emerging trends by continuously contrasting and comparing responses and as a result, this method offers flexibility for new themes to be formed, as well as initial themes to be collapsed or reclassified. This process encourages the translation of qualitative material into quantifiable results by providing a number or percent of how many participants endorse each category or theme. On average, 98% (*n* = 280) of participants responded to each open-ended question.

## Results

### Quantitative Analysis

Scale descriptive statistics as well as Pearson’s correlations between PTGI total scores and key predictor variables are presented in Table 2. Bivariate correlations were first conducted to determine the relationships between PTG, coping, perceived social support and psychopathy. Further, as evidence suggests that women may experience more PTG than men (e.g., Yilmaz & Zara, 2016), the correlation between biological sex and PTG was also examined. Results indicated that both approach coping (*r* = .29, *p* < .001, 95% CI [0.17, 0.39]) and psychopathy (*r* = .22, *p* < .001, 95% CI [0.10, 0.34]) were positively associated with PTGI scores, while PSS, avoidance coping, and sex were not (*p*’s > .05).

**Table 2.** Means, Standard Deviations, and Pearson’s Correlations Between PTGI Total Scores and Key Predictor Variables.

Scale	*M*	*SD*	Range	Correlations				
				PTGI	PSS	Av	Ap	SRP
PTGI	47.71	27.00	0–105	—				
PSS	26.83	8.03	3–36	.08	—			
Brief COPE								
Avoidance (Av)	25.25	5.94	12–44	.08	−.21*	—		
Approach (Ap)	34.47	7.03	12–48	.28*	.42*	.26	—	
SRP	87.08	18.60	58–138	.22*	−.05	.10	−.05	—

*Note. N* = 285. PTGI = The Posttraumatic Growth Inventory (Tedeschi & Calhoun, 1996); PSS = Perceived Support Scale (Kaniasty, 1988); SRP = The Self-Report Psychopathy Scale 4th Edition: Short Form (Paulhus et al., 2016).

* *p* < .001.

A series of hierarchical linear regressions were next conducted as a follow-up to the correlational analyses. Although PSS was not significantly correlated with PTGI scores, it was included in the final step of the regression as we had made a specific hypothesis that PSS would be related to increased PTG. As approach coping demonstrated the strongest correlation with PTGI, it was added in the first step, where it significantly predicted participants’ posttraumatic growth, *F*(1, 283) = 24.15, *p* < .001, *R*^2^ = .079. Psychopathy scores were added into the next step, where this new model remained significantly predictive of PTGI, *F*(2, 282) = 21.93, *p* < .001, *R*^2^ = .135. The addition of psychopathy scores improved the model fit significantly, Δ*F*(1, 282) = 18.24, *p* < .001, Δ*R*^2^ = .056. In this model, both approach coping and psychopathy were significant predictors of PTGI (*t*(282) = 5.28, β = 1.13, *p* < .001; *t*(282) = 4.27, β = .34, *p* < .001, respectively). PSS was added as the next step in the third and final model. Although this resulted in a significant model, *F*(3, 281) = 14.70, *p* < .001, *R*^2^ = .136, the improvement in model fit was not significant, Δ*F*(1, 281) = 0.33 *p* = .57, Δ*R*^2^ = .001. In this final model, both approach coping and psychopathy scores were significant predictors of PTGI *t*(281) = 5.04, β = 1.18, *p* < .001; *t*(281) = 4.25, β = .34, *p* < .001, respectively), while PSS was not (*t*(281) = 0.57, β = −.12, *p* = .57).

### Qualitative Analysis

Table 3 provides a brief summary of the results for each open-ended question including reoccurring themes and example sentences.

**Table 3.** Themes That Emerged Based on Responses to Questions on Support, Coping and Learning.

Theme	Frequency *n* (%)	Example quote(s)
Support received		
	*n* = 212	
Emotional	177 (84)	“. . ., helped me express my emotions”
Informational	47 (22)	“Some legal advice”; “they gave me advice”
Tangible	20 (9)	“My family. . . is actively networking for me for a new job”
Financial	8 (4)	“My family offered financial support so I could quit”
Spiritual	2 (1)	“I had a group of Christian friends I was praying with”
Coping method		
	*n =* 215	
Emotion-focused	84 (39)	“reduced my stress with exercise, friendships, massage”
Positive	69 (82)	“[seeking] help from family and friends”; “Exercise”
Negative	25 (30)	“I have started drinking a lot to take my mind off this”
Avoidance	76 (35)	“Avoid the person”; “I tried to ignore her”
Problem-focused	75 (35)	“Addressed my concerns with my supervisor, human resources and upper management”
Learning or growth		
	*n* = 214	
Positive personal growth	66 (31)	“I feel I have become an even stronger person”; “Emotional resilience”
Less trusting	53 (25)	“trust people less”
Enhanced identification	42 (20)	“I am more able to recognize that kind of person now”
Negative outcomes	24 (11)	“I’ve learned that life is awful”; “I live in fear”
Future responses	20 (9)	“. . . speak up to bosses”
Self-learning	11 (5)	“I’ve learned a lot about myself”; “now I know myself better”

*Note*. Percentages for each theme are based on the number of survivors who had indicated receiving support, using coping strategies, or learned/grew as a result of working with the alleged psychopath.

#### Type of support received

Overall, 74% (*n* = 212) of survivors endorsed receiving some type of support from friends, family or colleagues in dealing with the alleged psychopath, where five recurring themes emerged, including emotional, informational, tangible, financial, and/or spiritual support. Percentages within each theme are based upon the number of survivors that had indicated obtaining some type of support.

Although a small number of survivors (*n* = 26; 9%) out of the total sample explicitly indicated not receiving any support (e.g., “I had to deal with the situation alone”; “I had no real support system at the time”), survivors most commonly reported receiving one type of support (54%, *n* = 155). Fifteen percent (*n* = 44) mentioned two methods of support (e.g., “my partner and mother gave me space to talk about the effect he was having on me emotionally as well as giving me advice about how best to manage/avoid him”) and 1% (*n* = 3) specified receiving three methods of support.

I mainly have talked to my significant other about this issue and he has been helping me find another job so I don’t have to work with the psychopath anymore. He has also talked to my boss about the situation since my boss didn’t do anything about it when I talked to him. He also offered to help pay for my bills if I wanted to quit my current job while looking for another job. My significant other has been very supportive emotionally during this time. My good friend has also been very supportive and a great person to talk to during this difficult time.

##### Emotional support

The vast majority (84%; *n* = 177) of narratives denoted receiving emotional support which corresponded to any communication or understanding by which the survivor felt they had been heard or understood. Survivors who mentioned they received professional emotional support (e.g., psychologists, therapists) were not included in this category. Typical responses included, “I received support in the form of allowing me to vent my frustrations,” “My friends and family were there for me as I went through this ordeal. They listened to me, offered encouragement, supported me emotionally, [and] reassured me,” and “[Luckily] for me: a lot of emotional support from family and colleagues! A lot of understanding, concern and empathy.”

##### Informational support

The second most commonly endorsed theme was informational support (22%; *n* = 47), which most commonly took the form of advice or strategies on how to deal with the alleged psychopath: “Friends and family told me to ignore him, but when they saw how it was taking a toll on me, they suggested I denounce him to management or [human resources]”; “Some urged me to get legal help”; Work colleagues . . . have encouraged me to go through the grievance procedure in complaining about the bullying.”

##### Tangible support

Nine percent (*n* = 20) of survivors mentioned receiving tangible support or anything that the survivor was given, provided with or could use. Examples of tangible support included networking (e.g., “many community members have offered me networking opportunities or looked for work for me, trying to help me find employment”), aiding the survivor in their job search (e.g., “My colleagues have offered to be references or write letters of recommendations), assisting with various tasks (e.g., “My daughter helped me most by . . . helping me write formal complaints”), and/or being given trips or gifts (e.g., “They would often try to distract me with surprises, such as short weekend getaways or other fun things”).

##### Financial support

A small number of narratives (4%; *n* = 8) indicated receiving financial support or assistance, where typical responses included: “. . .gives me money for my needs,” and “My significant other . . . offered to help me pay for my bills if I wanted to quit my current job while looking for another job.”

##### Spiritual support

One percent (*n* = 2) of survivors mentioned receiving spiritual support from their social network. Responses included “spiritual support” and “I had a group of Christian friends I was praying with.”

#### Coping methods

In response to being asked how survivors dealt with the behaviors caused by the alleged psychopath, three primary themes occurred: emotion-focused, avoidance, and problem-focused. In total, 215 survivors (75%) reported utilizing some form of coping strategy, while 4% (*n* = 11) stated that they did not, had not, or didn’t know how they dealt with the behaviors caused by the alleged psychopath (e.g., “There was no way to deal with this situation”; “I did not deal with it at all”; “I did not know how to deal with it”). It is noteworthy to mention that while these survivors explicitly stated they did not deal with the situation, in their responses, three participants did indicate utilizing some form of coping method (e.g., “no idea . . . I try yoga, try to focus on my new job opportunities and I talk a lot”; “after explaining the situation to an employment [councilor] I was advised to find employment and then lodge a complaint with safety and health”). Percentages in each theme were constructed based upon on those who reported dealing with the behaviors caused by the alleged psychopath.

##### Emotion-focused coping

The most commonly endorsed (39%; *n* = 84) coping strategy was emotion-focused coping styles which involved taking steps to reduce negative emotional responses, included engaging in behaviors such as reframing the situation, using substances or medication, or seeking professional support (e.g., “Denial, crying, alcohol and drugs, talking to others, seeing a shrink”). Within the parent theme of emotional-focused coping, both negative and positive styles of emotion-focused coping emerged.

Positive strategies were the most commonly endorsed (82%, *n* = 69) sub-theme of emotion-focused coping, where survivors indicated talking through their emotions and feelings by seeking social support and/or professional help (e.g., “I went to a family therapist”; “Discussing it in detail with my trustworthy friends and family”; “Counselling, talking with my best friend, talking with my family”), engaging in mindfulness (e.g., “I now practice regular meditation”; “ I continued writing a journal”), and distraction with other activities (e.g., “Exercise! I had to recover and do sports”; “In the middle of the night, I would read or play computer games to avoid the worrying feeling”).

Negative strategies, which encompassed suppressing emotions by medication, alcohol or drug use, were endorsed by 30% (*n* = 25) of survivors, where typical responses included: “I definitely drank too much, . . . at one point in time, I took my colleague’s anti-anxiety medication in attempt to cope with some particularly terrible days,” “I took Xanax and sleeping pills,” “Denial, crying, alcohol and drugs,” and “I went on medication.”

##### Avoidance

Over one-third (35%; *n* = 76) mentioned engaging in avoidance or distancing behaviors, where survivors indicated actively evading, ignoring or limiting contact with the alleged psychopath. Typical responses in this theme were: “Avoid them at all cost,” “I did not go to work at all,” “I changed my position again to have somewhat less to do with him, and I avoid him whenever possible,” “Ignoring his emails, . . . avoiding physical contact at meetings,” and “I ignored the harassment as much as possible.”

##### Problem-focused coping

The final theme to emerge was problem-focused coping, which was endorsed by 35% (*n* = 75) of survivors. Problem-focused coping referred to when one actively attempted to take control of their stress or perceived stressors. Seeking information or education related to psychopathy and/or bullying was popular for many. Survivor responses included “I attended workshops on Workplace Bullying and Dealing with Difficult People,” “Education through university courses on Forensic Psychology has greatly assisted me in dealing with a Psychopath.” and

I also began to actively read everything I could find on psychopaths so that I would never again be a victim of one. I took an excellent workshop on manipulators that helped me understand how I was chosen to be his victim. Knowledge is power.

Several survivors also mentioned taking detailed steps in order to cope or deal with the problems caused by the alleged psychopath. Typical responses here often encompassed addressing the alleged psychopath directly, approaching human resources or other governing bodies, taking detailed logs and notes of their work and/or resigning from their position.

I tried to address the situation mutually between us . . . the approach was not successful. . . . I subsequently approached second-level management; and this led to a series of meetings with H.R. I made formal complaints including to H.R. I resigned my position.

Went to supervisor for clarification on work assignments and problems with reports. . . . I looked for another position within the department and got a better position a year after she was reassigned away from me. I kept a running log to show work tasks accomplished and provided a monthly report to the extended work group. I made a “deliverables” list for the psychopath at the direction of a higher-up. . . . I accessed my work Employee Assistance Program for practical advice. I talked to co-workers to get reality checks on “is it her or me?”. . . I sought legal advice when a work evaluation was poor due to her manipulation.

#### Learning or growth

When asked if survivors felt they had learned important lessons or had grown as a result of working with the alleged psychopath, 75% (*n* = 214) mentioned they had experienced some form of growth and/or learning. Six themes appeared including the following: positive personal growth, less trusting of others, enhanced identification, negative outcomes, future responses, and self-learning, where percentages within each theme were calculated according to the number of survivors who reported post-experiential growth or learning. This being said, 9% (*n* = 25) explicitly mentioned not exhibiting any form of growth or experiential learning (e.g., “absolutely not”; NO I live in fear”), nor was growth and learning a positive experience for all; some survivors mentioned negative effects as a result of their experience. One participant explained that while they did experience growth and learned valuable lessons, the emotional and physical toll was severe.

I have grown and learned very important lessons, but the price was very, very high: the erosion of my self-esteem and confidence, my physical health, emotional pain and financial devastation. I will never again be so naive as to trust people who set off my internal alarms. I will never again make excuses for bad behavior. I will never again trust easily, and I will RUN from anyone who displays psychopathic tendencies.

##### Positive personal growth

Nearly one-third (31%, *n* = 66) of survivors reported experiencing positive personal growth as a result of their encounters. Survivors often mentioned feelings of increased mental strength, resilience, adopting better coping skills, or being more appreciative of friends and family. Comments in this theme included the following: “I have grown to enjoy everyday as much as possible—to be thankful for everything—to appreciate and respect the lives of others,” “I am a stronger person than I once was,” “not to be afraid to lean on the ones around you when you truly need emotional support,” and

My family is extremely important, more than it was, and I take better care of my physical and mental health. I am happy that I found physical exercise ‘back’ as something that I can do in my (scarce) leisure time, but I do enjoy that a great deal.

##### Less trusting

Fifty-three participants (25%) reported being less trusting and more cautious, careful, and wary of others as a result of working with the alleged psychopath. Typical survivor responses were “. . .trust people less,” “don’t trust anyone,” and “be less trusting.” One participant in particular provided specific instances where they would be cautious of others in the future.

Be very careful with making friends at work, especially if you invite them/ they invite you to meet as friends outside of work. Be very wary of people who have made a lot of enemies but seem to be treating you as an exceptional friend. Be equally wary of them using excessive flattery. Note discrepancies in the person - contradictions between their espoused (caring) professional values and how they act/talk about colleagues and even service users.

##### Enhanced identification

The third most commonly referenced theme (20%, *n* = 42) was being better able to identify a bully or someone with psychopathic characteristics in the future. Survivors mentioned taking proactive steps to educate themselves on psychopathy and bullying, being more cognizant of and recognizing deception and/or manipulation, or by being more trusting of their gut feelings and instincts. Responses here included: “gained tremendous familiarity with behavioral ‘red flags’ signaling potential sociopathic/psychopathic individuals,” “I also seem to be better able to recognize manipulation. I now listen to and trust my instincts/intuition,” and “I can recognize the traits of a psychopath more easily and avoid these people immediately.”

##### Negative outcomes

Eleven percent (*n* = 24) of survivors stated suffering negative feelings and emotions (e.g., “I feel depressed and tired”; “I feel very ashamed,” “My life was a nightmare, felt powerless many times”), or negative impacts on their well-being as a result of their experience. One participant said, “I have gone from a giving teacher, passionate about her job [and] family to a waste of space,” while another reported, “I feel like I have lost faith in humanity,” and yet another wrote, “I’ve learned that life is often awful, with no apparent meaning or reason for it.”

##### Future responses

A handful of participants (*n* = 20; 9%) reported that they had learned how to proceed or respond to similar situations in the future, which included both proactive (e.g., “google your prospective boss”; “Do a background check. Get references.”) and reactive (e.g., “Speak up to bosses”; “Avoid them”) behaviors. Several survivors commented that they had realized the importance of setting boundaries and seeking options or alternatives to ensure that their mental health and well-being is not negatively impacted in the future.

I have realized that in this life, you need to put your foot down. You need to call it out when someone is treating you unfairly, and don’t think you are hurting someone else’s feelings back by doing this, or worry you are going to face repercussions.

I have also learned that I don’t have to put up with being treated badly at work. If the situation is hostile, as it was for me, I can get help and either try to cope with it, or move on to a new position. Life is too short to be stuck in a job that affects your health in a negative way. You should be able to enjoy your job so you can do the best that you can and make a difference.

##### Self-learning

A small number of survivors (*n* = 11; 4%) mentioned that they had learned a lot about themselves through their experience. Example responses in this theme include: “I have learned I do not function well in settings where I am not given control of my work,” “I understand myself better” and “I know myself better, my weaknesses, my vulnerabilities.” Notably, both positive (e.g., “I learned that I am more resilient then I thought”; “My health is the most important thing”) and critical (e.g., “But now I know myself better. Too naïve, too trusting, too needy”; “I learned I am less patient and forgiving than I thought”) self-learning occurred.

## Discussion

The present study sought to examine the potential positive outcomes of working with an individual exhibiting psychopathic characteristics in terms of posttraumatic growth (PTG) and reported well-being. Both psychopathy and approach coping styles were significant and unique predictors of PTG and survivors indicated utilizing a variety of coping skills and social supports, as well as undergoing a range of post-experiential learning and/or growth following their experiences.

It is noteworthy to mention that in our study, mean psychopathy scores corresponded to the 99.5th percentile in community samples and the 74th percentile in offender samples (Paulhus et al., 2016); this is attributable to the method in which psychopathy was measured. Despite the high scores potentially causing a ceiling effect, results indicated that higher psychopathy scores predicted greater PTG in survivors. Although counter to our prediction, this finding may actually reflect the degree in which one has come to understand and find meaning in their experiences (Calhoun et al., 2010). For this to occur however, one must actively think or ruminate about the experience holistically by considering the events themselves as well as the ensuing aftermath (Triplett et al., 2012). Two types of rumination have been proposed: deliberate rumination, when an individual intentionally thinks about an event, and intrusive rumination, when thoughts about the event occur spontaneously (Cann et al., 2011). Though intrusive rumination has been linked to heightened levels of posttraumatic stress (Taku et al., 2008; Triplett et al., 2012), it said to be the necessary precursor of deliberate rumination, which is positively associated with PTG (Cann et al., 2011; Shigemoto et al., 2017; Triplett et al., 2012). It’s plausible then, that those survivors who indicated that their colleague or supervisor had many psychopathic characteristics were ones that engaged in more deliberate thinking about their experiences, resulting in greater PTG. In fact, this was inadvertently addressed in written accounts when survivors described the positive personal growth they had experienced as a direct result of working with the alleged psychopath:

I feel that these encounters with psychopaths . . . have enhanced my emotional intelligence, my emotional control, made me understand what I value, made me aware of my intentions and be confident when others attempt to create self-doubt in me, and have made me acutely aware of how grateful I am for my own empathy and that I have for other deserving, loving people. I would not have had access to the joy that . . . loving myself and others gives me without having had the challenges of interacting with these people. . . . although I am grateful for the experience, I . . . now want to focus on the positive growth I have had.

To generate growth, some individuals may re-examine their relationships, beliefs, and priorities, while others may engage in techniques like cognitive reframing to substitute painful memories for positive ones (Aldwin et al., 1994). Certainly, with the majority of survivors indicating that they no longer worked with the alleged psychopath, positive reframing may have ensued where survivors attempted to replace their negative memories with more optimistic aspects of these same memories and experiences. In accordance with this, our results suggest that approach-focused coping is a significant predictor of PTG. This is in alignment with prior research where those who engaged in approach or problem-focused coping styles, including positive reframing, active coping, emotional support, and acceptance, were more likely to experience PTG following an adverse or traumatic event (e.g., Prati & Pietrantoni, 2009; Schmidt et al., 2011; Scrignaro et al., 2011; Thornton & Perez, 2006; Widows et al., 2005; Yilmaz & Zara, 2016). In their responses to the open-ended questions, survivors commonly reported utilizing problem-focused coping strategies by seeking social support from friends, family or colleagues, and took action to change their situations by addressing their concerns with upper management or human resources, seeking legal aid, or pursuing alternative job arrangements.

Some researchers posit that when individuals feel their situations are unchangeable or unsolvable, they will adopt avoidance coping (Webster et al., 2014; Yagil et al., 2011). Indeed, in the current study, survivors reported utilizing avoidance coping mechanisms including medicating or actively avoiding the alleged psychopath in attempts to reduce the emotional and physical harms they were enduring. In Webster et al.’s (2014) study examining the coping strategies used by 76 individuals to reduce the psychological and physical afflictions of working with a toxic leader, employees reported resigning from their positions once they believed their primary coping strategy to be ineffective. Although not overtly mentioned as a last resort, in the present study survivors may have sought new employment in response to feelings that all other possible options for reconciliation were exhausted. This is supported by many survivors indicating they left the organization following their many failed attempts to resolve the problems caused by the alleged psychopath:

I tried addressing issues with her directly as they came up. . . . I asked for changes in my work schedule. I brought my concerns to management and [human resources]. I made informal and formal complaints under the respectful workplace policy. . . . I asked for alternative dispute resolution. I involved the union. I made a public interest disclosure. I attended workshops on Workplace Bullying and Dealing with Difficult People. I contacted the Department of Labour, and the ministers of both Health and Labour. . . . I resigned.

Our prediction that perceived social support would be positively related to PTG went unsupported. Despite social support recurrently exhibiting links with PTG and resiliency (Layne et al., 2007; Leung et al., 2010; Prati & Pietrantoni, 2009; Thornton & Perez, 2006) some researchers have found the relationships between social support and PTG to be more complex. Schmidt et al. (2011) for example, found that availability of social support did not impact PTG in a sample of cancer survivors, while Scrignaro et al. (2011) results suggested that in the short-term (i.e., during traumatic or stressful event), perceived availability of support is significantly associated with PTG, but over time (e.g., 6 months following a traumatic or stressful event), perceived availability of support, actual support received, and satisfaction of received support is unrelated to PTG.

Although it is possible that perceived social support was not related to PTG due to the time elapsed between survivors working with the alleged psychopath and the time of survey, the fact remains that social support and connection appear to be significant contributors when individuals are faced with stressful, traumatic or unfavorable events. Survivors frequently mentioned social support in all aspects of their written responses, whether it be that they sought social support as a mechanism to cope with their circumstances, were detailing the type of social support they primarily received, or wanted to draw attention to the growing appreciation for friends and family members they developed, with many stating these relationships improved following their experiences.

Enhanced interpersonal relationships were not the only positive outcome in this study. Along with PTG, survivors indicated greater emotional resiliency, recognized the importance of (and how to set) boundaries, developed constructive coping skills, were more mindful, had gratitude, and found enjoyment in life. The consequences of working with an alleged psychopath were not always positive, however. Some survivors experienced a mix of both positive and negative outcomes:

Far less trusting; more cautious; less delusional about the integrity of peers (i.e., I used to believe most had some; now less so); share less with colleagues. Indeed, a lot of negative outcomes. On the positive: I’m (even more) resilient; and have reassessed the weight of my work life in my day to day enjoyment/focus - spend more time actively enjoying other activities (to enjoy them, and to lessen the impact of negative work experiences specifically); less willing to sacrifice personal views/time/efforts for work.

while others experienced only negative effects:

No. I have damaged self-esteem, 8 months of unemployment, no references, a vacated trust in the dependability of co-workers. I suffered a nervous breakdown, and I learned a new depth of depression. I am healing, but it has taken time. For you to suggest that ANYthing positive could come from this experience implies that you do not appreciate the intensity of this experience.

This is not to say that those who only reported negative outcomes could not experience PTG. In fact, this study, along with others (e.g., Prati & Pietrantoni, 2009; Schmidt et al., 2011), suggest that making targeted changes toward adopting more approach or problem-focused coping methods may enhance the likelihood of positive growth following stressful or traumatic events. This being said, it is important to note that experiencing mistreatment, bullying or victimization does not and should not fall upon the survivor but rather the perpetrator and perpetrator alone. Evidence suggests that trust, compassion, and empathy within the workplace leads to increases in productivity (Barsade & O’Neill, 2014), job satisfaction (Barsade & O’Neill, 2014), employee retention (Zak, 2017), and engagement (Miao et al., 2016). People also appear to prefer working alongside pleasant but less capable and knowledgeable individuals than individuals who are competent, but unfriendly or hostile (Casciaro & Lobo, 2005). As such, organizations may benefit both financially and reputationally in fostering an empathetic environment in addition to developing and enhancing empathy within its constituents (Nowack & Zak, 2020).

In their study of 81 organizations, Feser et al. (2015) noted that effective leaders demonstrated four qualities: were supportive of staff, sought different perspectives to problems and performance-related issues, operated with a strong results orientation to foster high-quality work outputs, and solved problems effectively by gathering and interpreting various forms of information. With this in mind, it may be appropriate for hiring teams to assess candidates according to features of empathy, and effective leadership and communication styles, while placing less emphasis on a candidate’s ability to speak eloquently or command a room.

### Limitations and Future Directions

Although this study has numerous strengths including contributing to our understanding of the effects of corporate psychopathy, the positive outcomes that may result from such an experience, and integrated a qualitative component which is able to enrich and provide deeper context than quantitative data alone, it is not without its limitations. First, this study used a cross-sectional approach and as such, we are unable to draw any causal or temporal conclusions. That is, if traumatic events cause PTG or if growth following a traumatic event is an innate ability for some remains unknown. Second, as participants self-selected to participate within this study, a selection or sampling bias may have occurred inhibiting generalizability of findings. Third, as participants completed the SRP 4:SF on behalf of someone else, it is possible that the scores are not an accurate representation of the degree of psychopathic characteristics actually present within the alleged psychopath as this method of assessment allows for study participants to attribute their own biases or interpretations to the psychopath’s behaviors. For example, Jones and Hare (2016) found Boddy’s PM-MRV scale was strongly related to a co-worker’s ratings of dislike for their supervisor. In addition, Caponecchia et al. (2012) reported that participants who had been bullied at work were more likely to rate their co-worker as having more psychopathic characteristics as compared to those who had not been bullied. This said, Brieman and Kosson (2018) found that women’s partner-reported psychopathy scores using the Self-Report Measure of Psychopathy III (SRP-III; Paulhus et al., 2011) were positively correlated to men’s psychopathy scores using the Psychopathy Checklist-Revised (*r* = .55, *p* < .001; Hare, 2003), while men’s self-reported psychopathy scores using the SRP-III were not (*r* = .27; *p* = .066). Fourth, although we excluded participants with low psychopathy scores to ensure we were sampling from our intended population (those who have worked with a colleague or supervisor with many psychopathic characteristics), it is possible that the restricted range of scores reduced effect sizes.^2^ Finally, resulting from a lack of clarity and/or context, some written responses were unable to be coded and subsequently were not grouped into any theme(s). Due to the nature of how qualitative data was collected in this study (i.e., survey format), participants were unable to further explain or clarify their responses.

While this study found that both approach-focused coping and psychopathy score were both unique and significant predictors for PTG, they only explained approximately 14% of the variance of PTG. As such, future research should examine how other potential correlates (e.g., age, gender, ethnicity, personality) impact growth following a stressful or traumatic event. Additionally, given the exploratory nature of the present research, replication studies are necessary to ensure reliability and validity of results, especially within diverse populations and corporations.

## Conclusion

Contextual pieces, as found within our study, provide a more robust knowledge basis around the range of consequences for those who are victimized by psychopaths in the workplace and are essential in informing public perception and in discovering ways to help survivors mitigate their difficulties resulting from victimization. Overall, results suggest that while there is ample opportunity for positive growth and learning following the experience of working with an alleged psychopath, we must not ignore that not everyone experiences, interprets, and responds to stressful or traumatic stimuli equally; following an adverse event or series of events, some may experience limited positive outcomes, while others may experience no positive effects at all. Attempting to create constructive work environments by fostering prosocial leadership behaviors, developing effective communication and problem-solving skills, and advocating for organizational and mental-health supports is crucial in the success and well-being of a corporation and its employees.

## Notes

1. Low psychopathy scores were used as an exclusionary criterion. For a detailed description of how psychopathy scores were categorized, see the Quantitative Analysis subsection of the Method.

2. Upon reanalyzing our quantitative data with the full sample (*N* = 346), we found that the pattern of results remained the same, with minimal increases in effect sizes.
